# Host defense peptides human β defensin 2 and LL-37 ameliorate murine necrotizing enterocolitis

**DOI:** 10.1016/j.isci.2024.109993

**Published:** 2024-05-15

**Authors:** Shiloh R. Lueschow-Guijosa, Amy H. Stanford, Jennifer N. Berger, Huiyu Gong, Timothy J. Boly, Benjamin A.H. Jensen, Peter Nordkild, Alexandra J. Leegwater, Jan Wehkamp, Mark A. Underwood, Steven J. McElroy

**Affiliations:** 1 Department of Pediatrics, University of Iowa, Iowa City, IA 52242, USA; 2 Department of Pediatrics, Children’s Minnesota, Minneapolis, MN 55404, USA; 3 Department of Biomedical Sciences, Faculty of Health and Medical Sciences, University of Copenhagen, 1165 Copenhagen, Denmark; 4 Defensin Therapeutics ApS, 2870 Gentofte, Denmark; 5 Department of Internal Medicine, University of Tübingen, 72074 Tübingen, Germany; 6 Department of Pediatrics, University of California Davis, Sacramento, CA 95616, USA

**Keywords:** Biological sciences, Molecular biology, Immunology

## Abstract

Necrotizing enterocolitis (NEC) is a leading cause of preterm infant morbidity and mortality. Treatment for NEC is limited and non-targeted, which makes new treatment and prevention strategies critical. Host defense peptides (HDPs) are essential components of the innate immune system and have multifactorial mechanisms in host defense. LL-37 and hBD2 are two HDPs that have been shown in prior literature to protect from neonatal sepsis-induced mortality or adult inflammatory bowel disease, respectively. Therefore, this article sought to understand if these two HDPs could influence NEC severity in murine preclinical models. NEC was induced in P14-16 C57Bl/6 mice and HDPs were provided as a pretreatment or treatment. Both LL-37 and hBD2 resulted in decreased NEC injury scores as a treatment and hBD2 as a pretreatment. Our data suggest LL-37 functions through antimicrobial properties, while hBD2 functions through decreases in inflammation and improvement of intestinal barrier integrity.

## Highlights

• Host defense peptides LL-37 and hBD2 reduce NEC-like murine injury
• LL-37 acts through antimicrobial and wound-healing mechanisms
• HBD2 acts through immunomodulation and wound healing mechanisms
• HBD2 has a better biosafety profile than LL-37

## Graphical abstract

**Figure:**
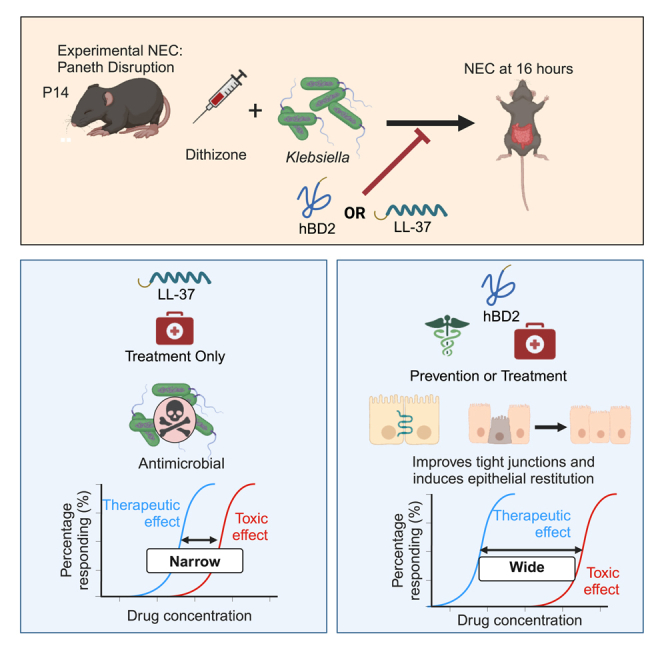

## Introduction

Preterm birth is the leading cause of neonatal morbidity and mortality^1^^,^^2^^,^^3^^,^^4^ and significantly impacts 15 million infants globally each year.^4^^,^^5^^,^^6^ Preterm delivery results in underdevelopment of most organ systems, but of particular interest is the intestine, which is the largest surface of the body that interacts with the outside environment.^6^^,^^7^^,^^8^^,^^9^^,^^10^ Intestinal immaturity is thought to partially explain why preterm infants are uniquely susceptible to certain diseases such as necrotizing enterocolitis (NEC).^11^^,^^12^^,^^13^

NEC is a devastating gastrointestinal disease resulting in severe inflammation and patchy to total intestinal necrosis.^14^^,^^15^^,^^16^^,^^17^^,^^18^^,^^19^^,^^20^^,^^21^^,^^22^ While the specific cause for NEC has yet to be elucidated, three major risk factors have been identified including prematurity, feeding, and microbial dysbiosis.^15^^,^^19^^,^^20^ In addition to short term morbidity and mortality, NEC survivors often encounter multiple long-term morbidities such as intestinal strictures, short bowel syndrome, and poor neurodevelopment.^15^^,^^23^ Despite decades of research, treatment strategies for NEC remain limited and non-targeted, including broad spectrum antibiotics, cessation of oral feedings, and operative therapy to remove necrotic bowel.^15^^,^^24^ Therefore, there is a desperate need for better treatment and, ideally, prevention strategies to manage NEC. One possible solution is the use of host defense peptides (HDPs). HDPs are small cationic peptides widely conserved in nature. According to the Data Repository of Antimicrobial Peptides (DRAMP) Database, there are currently over 6000 identified natural and synthetic known AMPs.^25^ While HDP’s function as direct antimicrobials through disruption of cellular membranes, they also can influence the host immune system and epithelial homeostasis.^26^ This diverse functionality allows HDPs to act as a first line of mucosal defense in the intestinal tract against pathogens.^26^^,^^27^^,^^28^^,^^29^^,^^30^^,^^31^

The mammalian small intestine expresses two families of endogenous HDPs, the cathelicidins and defensins.^31^^,^^32^^,^^33^ Humans and mice each express only one endogenous cathelicidin peptide. The cationic antimicrobial peptide (hCAP18) or LL-37 is found in humans while mice express a homologue of LL-37 named cathelicidin-related antimicrobial peptide or CRAMP.^34^^,^^35^ LL-37 has broad spectrum antimicrobial activity, binds and neutralizes lipopolysaccharide, stimulates cytokine release and chemotactic activity, promotes wound healing and angiogenesis, and modulates apoptosis.^31^^,^^34^^,^^36^^,^^37^ While use of LL-37 in NEC has not been studied, multiple studies have demonstrated that the administration of exogenous LL-37 in animal models of sepsis decreases the overall illness severity, lowers serum inflammatory cytokine levels, and improves survival rates.^35^^,^^38^^,^^39^ In addition, mice deficient in CRAMP are more susceptible to infections.^40^^,^^41^^,^^42^ However, a known issue with use of LL-37 is a dose-dependent toxicity at higher doses which was also seen in prevention of sepsis in animal models.^35^^,^^38^^,^^39^

The second family of HDPs found in the intestines of humans and mice is the defensins which also play an important role in intestinal innate immunity and may hold more therapeutic promise.^43^ Humans produce two types of defensins: α- and β-defensins. While α-defensins are commonly found in secretory cells such as Paneth cells and neutrophils, β-defensins are mainly expressed in epithelial tissues.^32^^,^^44^^,^^45^^,^^46^ Of note, human beta-defensin 2 (hBD2) was the first inducible human HDP discovered and was initially isolated from psoriatic skin lesions.^47^ hBD2 plays an important role in gut mucosal immunity as it is expressed by epithelial cells throughout the gut, has a broad spectrum of antimicrobial activity, and influences several inflammatory signaling pathways.^48^^,^^49^ Expression of hBD2 is induced by inflammation and bacterial exposure and the alteration of hBD2 expression has been implicated in diseases of the human intestine.^32^^,^^50^^,^^51^^,^^52^^,^^53^^,^^54^^,^^55^ Koeninger et al. showed that systemically administered recombinant hBD2 was safe and well tolerated, mitigated inflammation, and improved the disease activity index when compared to standard treatments in three distinct animal models of adult inflammatory bowel disease. This raises the possibility of using hBD2 therapeutically.^56^ However, applications of hBD2 have mainly been examined in adult diseases, and the impact of hBD2 on the developing intestine is relatively unexplored.

Because of their dynamic interactions in host defense, HDPs such as LL-37 and hBD2 are intriguing options as treatment and prevention strategies for inflammatory diseases such as NEC. Thus, the objective of this study was to investigate the preventive and therapeutic impact of LL-37 and hBD2 on the intestinal injury associated with experimental NEC.

## Results

### Oral hBD2 treatment is well tolerated and does not induce significant inflammation or severe small intestinal injury in neonatal mice

**Figure 1:**
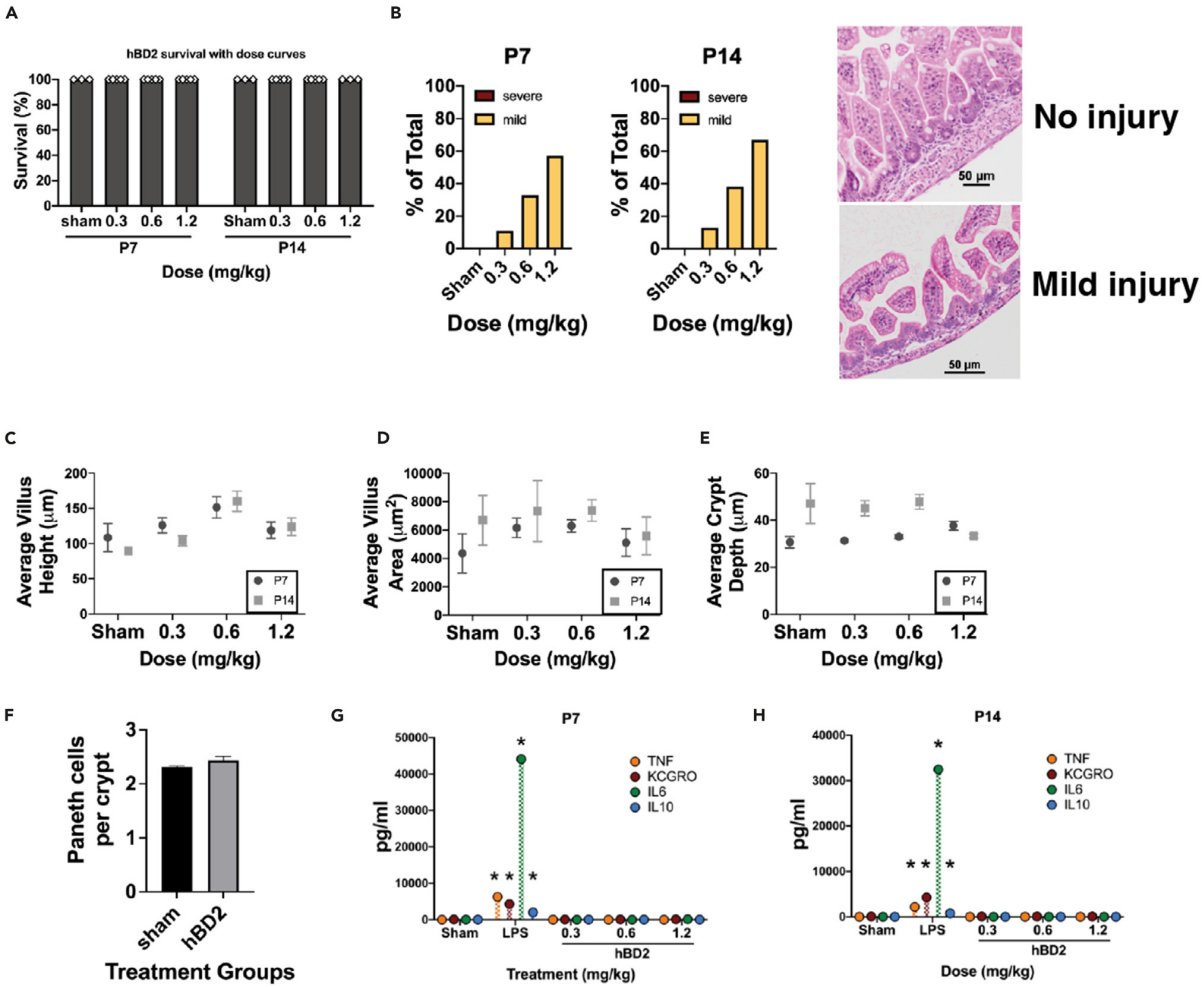
Oral hBD2 treatment at increasing doses does not induce inflammation or small intestinal injury in neonatal mice No mortality was seen in either P7 or P14 (n = 3–5 in all groups at both ages) C57Bl/6J mice following oral gavage of hBD2 at increasing doses as compared to sham (A). P7 and P14 C57Bl/6J mice were given hBD2 at increasing doses and their small intestine was evaluated for generalized intestinal injury. Injury was scored as either no injury (score of 0), mild intestinal injury (score of 1 representing mild separation of the lamina propria and/or mild villus vacuolization), or severe intestinal injury (score of 2 representing significant villous vacuolization, mucosal ulceration, lamina propria damage and/or presence of hemorrhage within villi) and compared to sham. No significant differences were seen in mice treated with hBD2 compared to controls. The percentage of mild intestinal injury for each dose is denoted by yellow bars. No animal sustained severe injury (B). Example histology of intestinal injury is shown. No significant differences were seen in combined villus height (C), combined villus area (D), or combined crypt depth (E) (*n* = 6–10 mice per group for all histologic measurement with 300 villi or 100 crypts measured per animal). Mice treated with hBD2 had no significant change in Paneth cell numbers per crypt compared to shams (2.4 vs. 2.3) (F). Serum was collected from P7 (G) and P14 (H) mice with increasing doses of hBD2 and quantified for TNF, KCGRO, IL6, and IL10 compared to both sham and non-lethal LPS treatments (0.01 mg/kg bw) as a positive control. (*n* = 3–5 per group, ∗*p* < 0.001). Only LPS treatments were significantly different from sham controls. Error bars represent SEM.

Murine dosing curves have been well established for exogenous LL-37, but dosing of hBD2 remains unknown. While Koeninger et al. showed that systemically administered recombinant hBD2 was safe and well tolerated in mice, they only looked at adult animals.^56^ Because hBD2 has not been studied extensively in neonatal mice, hBD2 was given at increasing doses from 0.3–1.2 mg/kg body weight (bw) twice daily for two days via gastric gavage to mice at P7 and P14. Animals were examined for survival, intestinal injury, microbiome, and serum cytokines levels. While treatment with hBD2 induced a dose-dependent mild intestinal injury, it had no significant impact on survival and did not induce severe injury at any dose (Figures 1A and 1B). Injury scores were determined as previously described.^57^^,^^58^^,^^59^ To examine if hBD2 treatment altered the small intestinal architecture, we measured the average villus height, villus area, and crypt depth of the small intestine. Treatment with hBD2 at P7 or P14 did not significantly change any measurement at any dose for either age (Figures 1C–1E). To make sure hBD2 was not impacting Paneth cell numbers, mice were treated with hBD2 and compared to sham controls. Treatment with hBD2 had no impact on the number of Paneth cells per crypt (Figure 1F). Treatment with hBD2 also did not majorly alter the microbiome of mice compared to sham controls. At both P7 and P14, no dose of hBD2 given to the mice had a significant impact on the bacterial phyla in the microbiome (Figure S1A). Similarly, when exploring the bacterial families in the microbiome, at P7 no significant changes were observed based on dose of hBD2 given (Figure S1B). More bacterial families were observed in P14 mice (Figure S1C). While some minor increases were observed in mice given 0.6 mg/kg bw hBD2 for Pasteurellaceae and Verrucomicobiaceae, there were no other notable differences observed for P14 mice when given any concentration of hBD2. There were also no clinically relevant changes in the serum cytokines TNF, KCGRO (murine equivalent of CXCL1), IL-6, or IL-10 when hBD2 was given via gastric gavage at either age when compared to what is seen after exposure to a non-lethal single dose of *E*. *coli* derived lipopolysaccharide (LPS, 0.01 mg/kg bw). (Figures 1G and 1H).

### LL-37 and hBD2 treatments reduce experimental NEC

**Figure 2:**
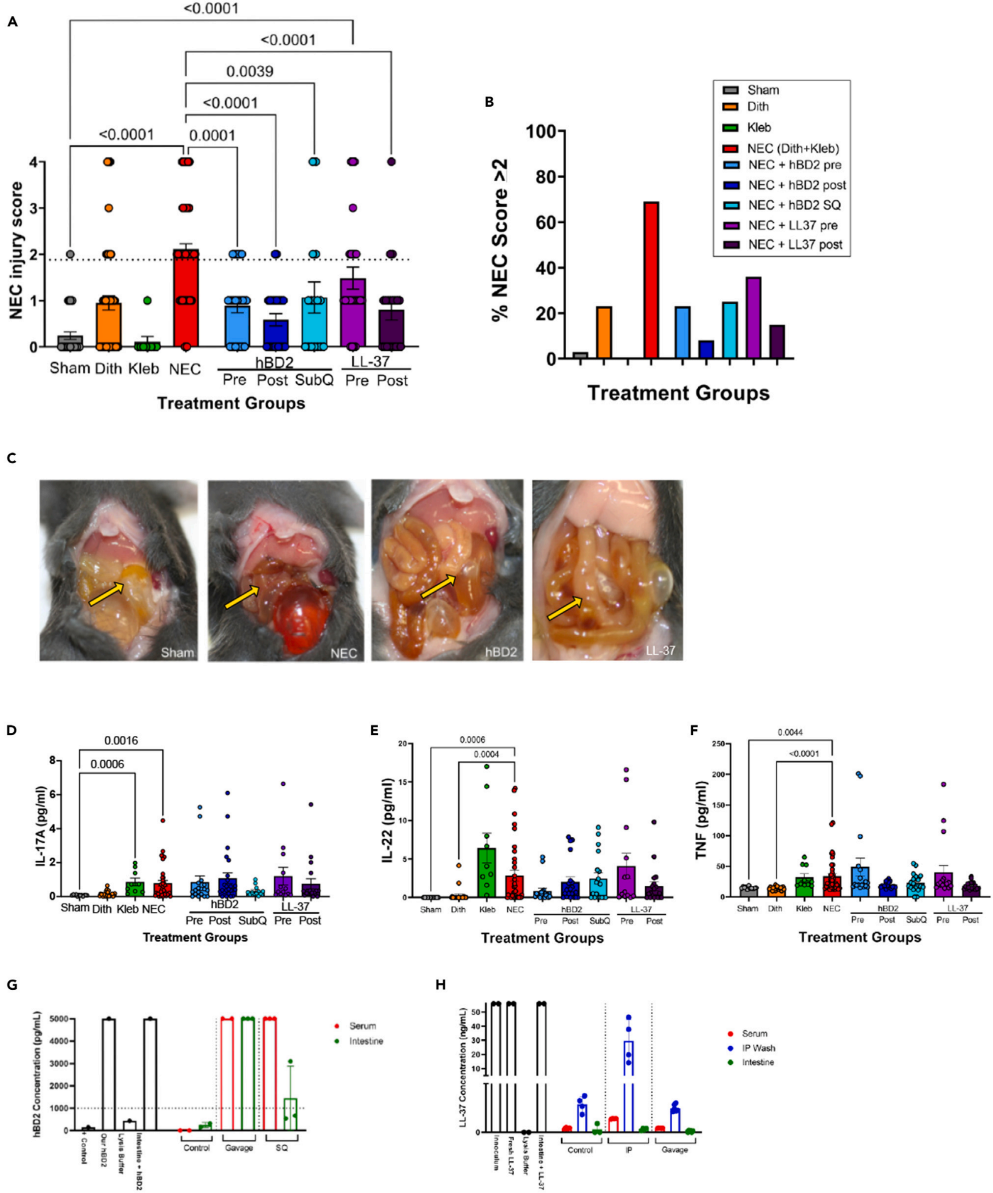
LL-37 and hBD2 significantly reduce experimental NEC induced by Paneth cell disruption with bacterial dysbiosis P14-P16 C57Bl/6J mice were treated with dithizone and *K. pneumoniae* to induce NEC. Following euthanasia, ileal samples were harvested and scored for NEC-like injury. NEC injury scores of sham (gray), Dithizone (Dith) (orange), *K. pneumoniae* (Kleb) (green), NEC (red), hBD2 pre (light blue), hBD2 post (dark blue), hBD2 subQ (aqua), LL-37 pre (purple), and LL-37 post (plum) are shown with the dotted horizontal line indicating a NEC like injury (score of 2 or greater). Each circle represents a single animal. Mice with NEC had increased injury compared to sham, hBD2 pre, hBD2 post, hBD2 subQ, and LL-37 post (n = sham: 37, dithizone: 60, *Klebsiella*: 9, NEC: 99, hBD2 pre: 26, hBD2 post: 24, hBD2 subQ: 17, LL-37 pre: 25, LL-37 post: 20; *p* < 0.004 for all) (A). Total percentage of animals developing significant disease (score of ≥ 2) (B). On necropsy, mice with NEC (second panel) had dark discolored intestines with adhesions, while animals treated with hBD2 or LL-37(third and fourth panels) had healthier appearing intestines and less adhesions that were more similar to sham conditions (first panel). Yellow arrows indicate small intestine (C). Serum levels of IL-17a, IL-22, and TNF were quantified at time of tissue harvest (D–F). Serum levels of IL17A, IL-22, and TNF were significantly elevated in NEC compared to sham (*p* = 0.0016, 0.0006, and 0.0044 respectively), but hBD2 and LL-37 treatment/prevention did not significantly alter any cytokine levels compared to NEC. P14-P16 C57Bl/6J mice were euthanized 30 min following SQ injection or oral gavage with hBD2 or oral gavage or intraperitoneal (IP) injection with LL-37. Serum and homogenized intestinal samples were obtained and quantified for hBD2 (G) and serum, homogenized intestinal samples, and intraperitoneal wash (IP Wash) samples were obtained and quantified for LL-37 (H). Intestinal homogenates from SQ treated mice had hBD2 levels >1000 pg/mL, while all other hBD2 treated samples had hBD2 levels that exceeded the detectable limit by the ELISA plate (*n* = 3 animals per group). Elevation of LL-37 was detected in the IP wash of IP injected animals, but elevation was not detected in the serum or intestine in LL-37 IP injection or gavage (*n* = 3 animals per group). Error bars in all figures represent SEM.

We next evaluated the ability of hBD2 and LL-37 to attenuate NEC-like injury in a murine NEC model developed by our laboratory. P14-P16 C57Bl/6J mice were given a two-hit insult of dithizone-induced Paneth cell disruption, followed by induction of intestinal dysbiosis through enteral exposure to *Klebsiella pneumoniae* as we have previously described.^60^^,^^61^ Intestinal histology was scored by a blinded investigator using a standard 5-point scoring system where scores of two and higher are considered NEC-like injury (Figures 2A and 2B).^57^^,^^60^^,^^61^^,^^62^ Although mice exposed to LL-37 as a preventative had a trending although non-significant reduction in injury compared to NEC mice, mice that received LL-37 as a treatment experienced significantly less injury compared to NEC mice (*p* < 0.0001). More convincingly, hBD2 resulted in significantly decreased injury scores (*p* ≤ 0.0001), whether provided as a preventative or therapeutic treatment.

In addition to oral delivery, hBD2 has been shown to be efficacious through subcutaneous (SQ) routes.^56^ To determine if SQ delivery would have similar efficacy against experimental NEC, C57Bl/6J mice were given 0.3 mg/kg bw of hBD2 via a subcutaneous injection in the nape of the neck 1 h after NEC induction. This dosing has been shown to be effective in experimental inflammatory bowel disease.^56^ Similar to what was seen with oral hBD2, C57Bl/6J mice given SQ hBD2 after induction of NEC had significantly decreased injury scores compared to the NEC alone group and no difference in injury scores compared to control groups (Figure 2A).

Strikingly, treatment with hBD2 or LL-37 improved the gross appearance of the intestine on necropsy compared to those exposed to NEC induction alone. C57Bl/6J mice in the sham group had pale and yellow appearing small intestines (yellow arrow) without adhesions, while the mice exposed to experimental NEC had grossly dark inflamed small intestines with numerous adhesions. Mice treated with either hBD2 or LL-37 had less discoloration and markedly less adhesions when compared to the NEC group (sample gross appearance in Figure 2C). In addition to intestinal injury, we examined serum levels of several NEC associated cytokines at the time of tissue harvest (Figures 2D–2F). Animals exposed to NEC induction had significantly higher levels of IL-17A (*p* = 0.0016, Figure 2D), IL-22 (*p* = 0.0005, Figure 2E), and TNF (*p* = 0.0039, Figure 2F), compared to sham controls. Neither prophylactic nor therapeutic hBD2 or LL-37 treatment was able to significantly ameliorate the increases in IL-22, TNF, or IL-17A.

Since subcutaneous dosing with hBD2 resulted in similar reduction of NEC as oral gavage, we next wanted to determine whether hBD2 was moving systemically throughout the body or localizing in the intestine depending on the route of delivery. To determine, healthy P14-P16 mouse pups were given an oral gavage or subcutaneous injection with hBD2, and the serum and intestinal tissue were harvested 30 min later. The measured concentration of hBD2 in both the serum and intestinal tissue homogenates with both hBD2 delivery types was greater than 1000 pg/mL suggesting systemic activity (Figure 2G). Similar to hBD2, we also wanted to determine where LL-37 was localizing following oral gavage. P14-P16 mouse pups were given an oral gavage of LL-37 and as a comparison, we gave an intraperitoneal (IP) injection. Thirty minutes following delivery of LL-37, serum, intestinal tissue, and intraperitoneal washes were harvested. While oral gavage and subcutaneous injection of hBD2 highly increased hBD2 detection in the serum and intestine, LL-37 did not result in increases of LL-37 detected in serum or intestine over control levels (Figure 2H). In contrast, IP delivery of LL-37 did result in higher levels of LL-37 detected in the IP wash and slightly higher levels in the serum although no difference was detected in the intestine (Figure 2H).

### Reduction of experimental NEC *in vivo* by hBD2 and LL-37 treatment is not primarily via detectable alterations of the microbiome

**Figure 3:**
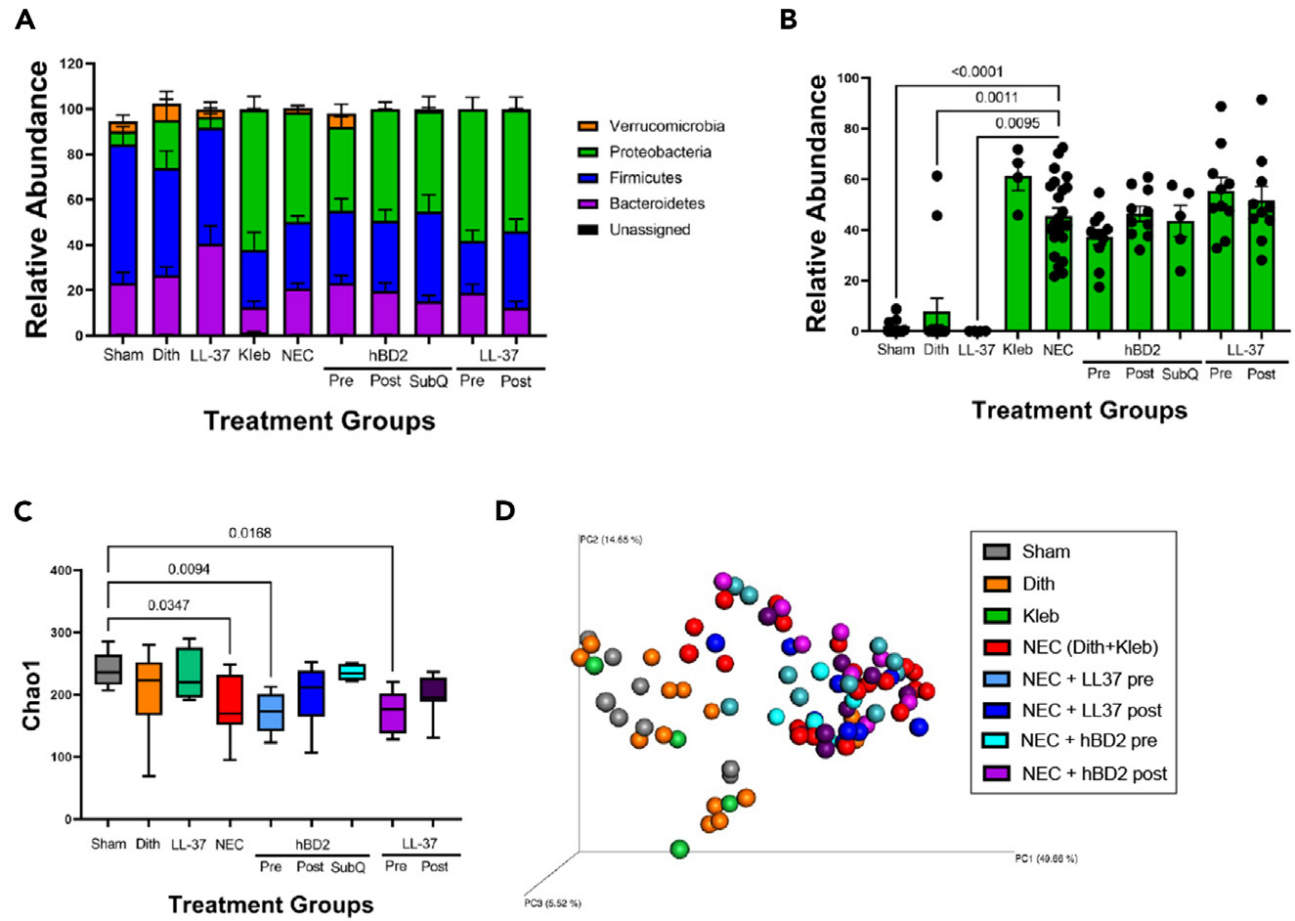
LL-37 and hBD2 prevention and treatment approaches do not cause detectable alterations in the cecal microbiome Cecal samples were analyzed for microbial composition using 16S sequencing. Phylum and family level analysis showed that induction of NEC altered the microbial composition by increasing the relative amount of Proteobacteria (*p* < 0.0001), and in particular Enterobacteriaceae (*p* < 0.0001), while decreasing the relative amount of Firmicutes. hBD2 and LL-37 treatment (pre- or post-induction of NEC) had no impact (n = sham: 6, NEC: 9, hBD2 pre: 11, hBD2 post: 10, hBD2 subQ 5, LL-37 pre 6, LL-37 post 5, *p* < 0.05) [(A): Phyla, (B): Enterobacteriaceae only)]. Alpha diversity analysis via chao1 demonstrated decreases in diversity in NEC and HDP pre-treated animals (C). Principle coordinate analysis of the 16S cecal microbiome indicates animals exposed to *K. pneumoniae* gavage (*Klebsiella*, NEC, pre hBD2, post hBD2, subQ hBD2, pre LL-37, and post LL-37) clustered together independent of HDP treatment and separate from non-*Klebsiella* animals (sham, dithizone) (D). Error bars in all figures represent SEM.

As antimicrobial peptides, we next wanted to ascertain what impact hBD2 and LL-37 were having on the core microbiome as a potential mechanism of action for the reduction in NEC injury. First, we examined the cecal microbiome composition through 16S rRNA gene sequencing. At the phylum level, the microbiome of mice receiving *K. pneumoniae* (Kleb, NEC, Pre hBD2, Post hBD2, SubQ hBD2, Pre LL-37, and Post LL-37) was marked by significant increases in Proteobacteria and compensatory decreases in Firmicutes compared to sham mice (Figure 3A). When looking more specifically at the family level, the increases in Proteobacteria were directly related to significantly increased Enterobacteriaceae, the family to which *K. pneumoniae* belongs (Figure 3B). Although hBD2 and LL-37 are both HDPs, neither hBD2 nor LL-37 significantly impacted the microbiome composition compared to NEC induction alone. We further examined the alpha diversity and compared to sham mice, NEC mice and NEC mice pretreated with hBD2 and LL-37 had significantly reduced alpha diversity (Figure 3C). Interestingly, NEC mice treated with LL-37 (sham vs. LL-37 *p* = 0.4414; NEC vs. LL-37 *p* > 0.9999) or hBD2 (sham vs. hBD2 *p* = 0.7907; NEC vs. hBD2 *p* > 0.9999) as post-induction treatments and, to a greater degree, subcutaneous injection of hBD2 (sham vs. hBD2 *p* > 0.9999; NEC vs. hBD2 *p* = 0.0841) had a non-significant trend toward higher alpha diversity scores than mice induced with NEC alone and were not significantly different from sham mice (Figure 3C). Next, we examined the beta diversity, which depicted that all NEC mice, regardless of delivery of antimicrobial peptide, clustered together, but distinctly from mice that did not experience NEC induction (Figure 3D).

### While LL-37 can directly kill *K. pneumoniae*, hBD2 reduction of experimental NEC is not primarily via antimicrobial effects toward *K. pneumoniae*

**Figure 4:**
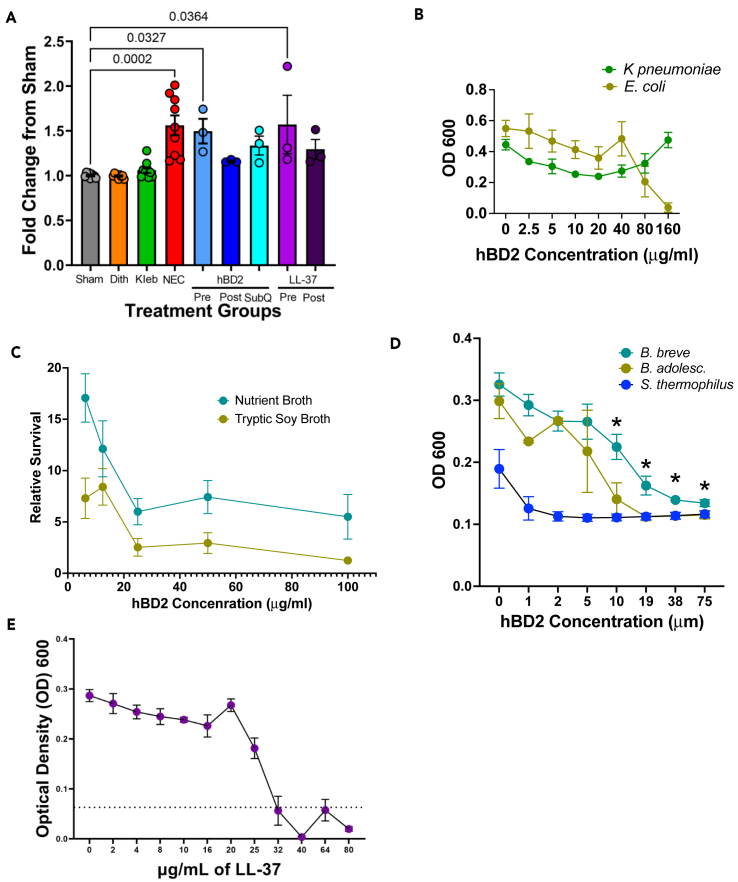
LL-37 can directly kill *K pneumoniae* 10031, while hBD2 has more limited antimicrobial capacity *K. pneumoniae* abundance was measured by PCR (A). NEC treatment significantly increased detection of *K. pneumoniae* (*p* = 0.0002, *n* = 9). Pre-treatment with HDPs LL-37 and hBD2 had no effect on NEC-induced increase in *K. pneumoniae* (*p* > 0.9999, *n* = 3), while post-treatment with HDPs LL-37 and hBD2 reduced *K. pneumoniae* to sham-levels (sham vs. Post LL-37 *p* = 0.1648; sham vs. Post hBD2 *p* = 0.8622 *n* = 3). Similar to oral therapeutic treatment with HDPs, SQ hBD2 decreased NEC-induced increases in *K. pneumoniae* toward sham levels (*p* = 0.1501). *K. pneumoniae* (6.3 × 10^6^ - 1.1 × 10^7^ CFU/mL) and *E. coli* (2.25 × 10^6^ - 1.585 × 10^7^ CFU/mL) were exposed to increasing concentrations of hBD2. hBD2 decreased growth for *E. coli* at 80 μg/mL, and achieved a MIC at 160 μg/mL, but no concentration of hBD2 significantly inhibited growth of *K. pneumoniae* (n = 2.5 μg/mL: 3, 160 μg/mL: 6, and all other doses 9) (B). *K. pneumoniae* grown in Nutrient Broth or Tryptic Soy Broth were exposed to increasing concentrations of hBD2. hBD2 induced a sharp decline in survival at concentrations of 25 μg/mL in both media types, but complete killing of *K. pneumoniae* was not observed in either media or at any concentration of hBD2 tested (*n* = 10 replicates for all strains and all doses) (C). In a turbidity assay, hBD2 caused significantly decreased bacterial growth for *Bifidobacterium breve* and *Bifidobacterium adolescentis*, but not in *S. thermophilus* (D). *K. pneumoniae* (5.15 × 10^6^ - 1.18 × 10^7^ CFU/mL) was exposed to increasing concentrations of LL-37 (E). LL-37 inhibited growth of *K. pneumoniae* at 25 μg/mL and completely inhibited growth at 32 μg/mL. Error bars in all figures represent SEM.

Based on the cecal microbiome data, hBD2 and LL-37 did not seem to influence the microbiome. However, since our NEC model is dependent on *K. pneumoniae*-induced inflammatory dysbiosis and both hBD2 and LL-37 possess antimicrobial activity, we wanted to determine the efficacy of hBD2 and LL-37 killing our specific strain of *K. pneumoniae*. PCR was performed using *K. pneumoniae* specific primers on small intestinal ileal tissues harvested at the end of the NEC model^63^ (Figure 4A, representative blots in Figure S2). While there were no significant differences in Proteobacteria or Enterobacteriaceae based on the 16S rRNA sequencing shown in Figure 3, based on the *K. pneumoniae* PCR, NEC animals given oral or subcutaneous therapeutic hBD2 or therapeutic oral LL-37 all had trending decreases in the amount of *K. pneumoniae* toward sham levels and were non statistically different from sham. In contrast, mice given oral pretreatment of hBD2 or LL-37 had significantly more *K. pneumoniae* detected compared to sham and comparable levels to what was observed in the NEC mice (Figure 4A). As a more direct measure of the effect of hBD2 and LL-37 on bacteria, minimum inhibitory concentration (MIC) and turbidity assays were performed (Figures 4B–4E). Interestingly, no concentration of hBD2 tested was able to significantly reduce the growth or kill our lab strain of *K. pneumoniae* (6.3 × 10^6^ - 1.1 × 10^7^ CFU/mL). To ensure functionality as an antimicrobial peptide, hBD2 was tested with *E. coli* (2.25 × 10^6^ - 1.585 × 10^7^ CFU/mL), which showed that at 80 μg/mL hBD2, growth was reduced and at 160 μg/mL hBD2, *E. coli* was completely inhibited (Figure 4B). Past research has shown that hBD2 is effectively able to kill *K. pneumoniae*, however, our data showed the opposite (Figure 4B). To resolve this discrepancy, we examined hBD2’s ability to kill *K. pneumoniae* using both Nutrient Broth (NB, recommended media for optimal growth of our *K. pneumoniae* strain and used in our laboratory for culture), and Tryptic Soy Broth (TSB, media used in data where hBD2 was cytotoxic to *K. pneumoniae*). Our data demonstrate that hBD2 is more effective at killing *K. pneumoniae* grown in TSB than *K. pneumoniae* grown in NB (Figure 4C). However, while hBD2 induced a decline in survival at concentrations of 25 μg/mL in both media types, complete killing of *K. pneumoniae* was not observed in either media or at any concentration of hBD2 tested. To further examine hBD2’s antimicrobial properties, a turbidity assay was next performed on common commensal bacteria. The data demonstrated that hBD2 exposure decreased the growth of two different bifidobacteria, but not a streptococcal strain (Figure 4D). Together, these results suggest that while hBD2 has antimicrobial properties against common gut commensal microorganisms and some Enterobacteriaceae species, hBD2 had limited ability to impact our lab’s strain of *K. pneumoniae* when utilized in an isolated, *in vitro* setting. We also wanted to determine the antimicrobial capacity of LL-37 against our lab strain of *K. pneumoniae.* In contrast to hBD2, LL-37 was able to reduce the growth of *K. pneumoniae* at 25 μg/mL and completely inhibited growth at 32 μg/mL (Figure 4E).

### Impact of hBD2 on NEC injury scores and cytokines depends on the NEC induction method

**Figure 5:**
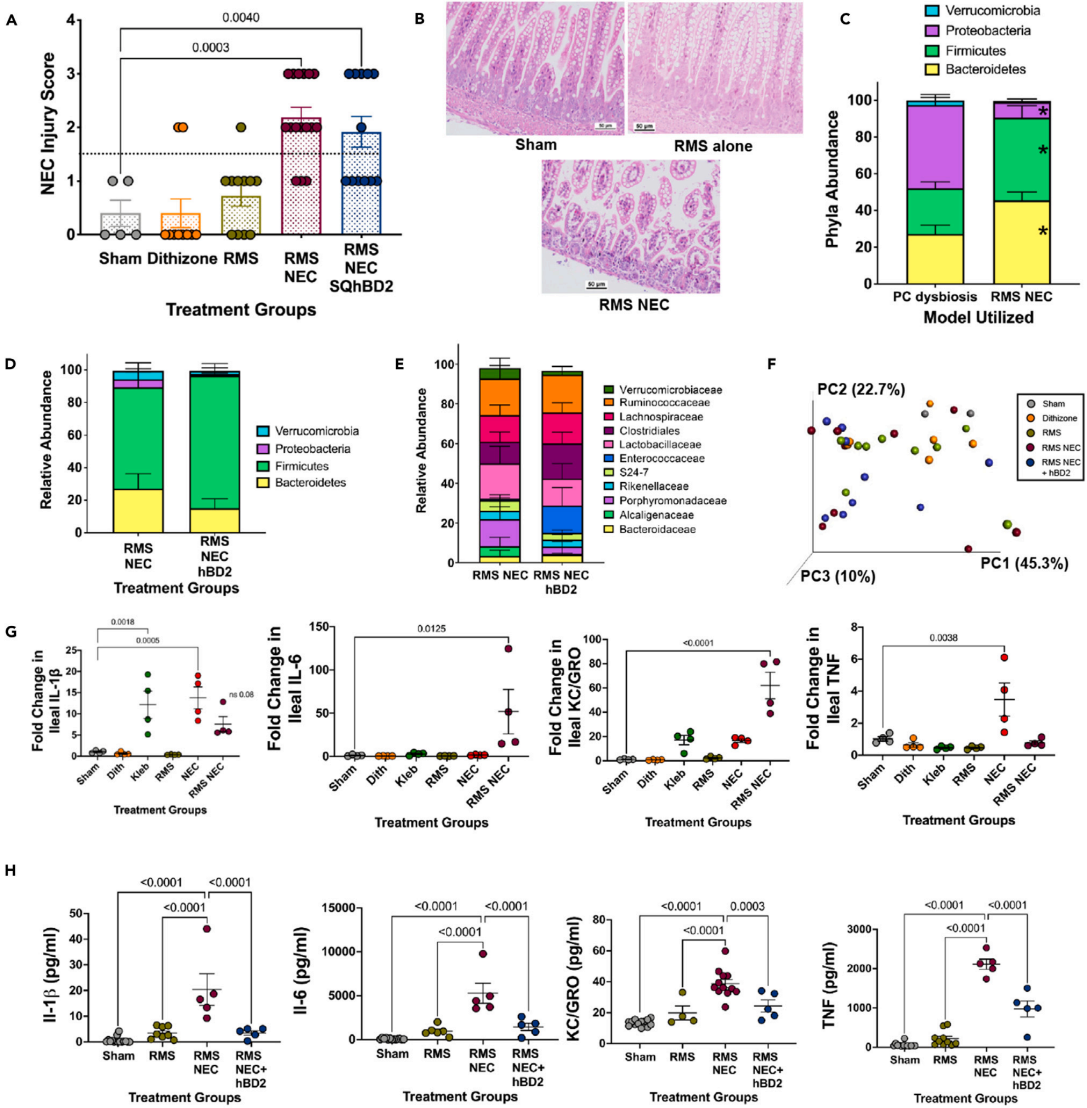
Impact of hBD2 on NEC injury scores and cytokine levels is NEC model dependent Experimental NEC independent of *K. pneumoniae* was induced in P14-P16 C57Bl/6J mice using RMS formula feeds and dithizone injection. Exposure to RMS formula feeds and Paneth cell disruption induced significant NEC-like injury compared to controls (*p* = 0.0003); however, hBD2 treatment did not prevent RMS NEC-induced injury (*n* = 10–15 per group) (A). Representative histology from groups (B). Paneth cell disruption with bacterial dysbiosis NEC induction of injury is associated with significantly greater relative abundance of Proteobacteria and decreased relative abundance of Firmicutes and Bacteroidetes species compared to Paneth cell disruption with RMS NEC induced injury (*n* = 7 Paneth NEC, 5 RMS NEC, *p* = 0.01 Bacteroidetes, 0.007 Firmicutes, and <0.0001 Proteobacteria) (C). Microbial composition of the cecum showed no significant differences between RMS NEC and RMS NEC treated with SQ hBD2 at either the phylum (D), or the family (E) levels, and had no significant clustering in Beta diversity (F) (*n* = 9 for all groups). Ileal samples were harvested, and gene expression was quantified. While the Paneth cell disruption with bacterial dysbiosis NEC model induced an upregulation of IL-1b and TNF genes, the Paneth cell disruption with RMS NEC model induced IL-6 and KCGRO genes (*n* = 3–4 per group, *p* values as shown) (G). Serum cytokine levels of IL-1b, IL-6, KCGRO, and TNF were all significantly increased after induction of NEC through the RMS NEC model. Treatment with hBD2 significantly blocked the RMS NEC-induced upregulation (*n* = 4–10 per group, *p* values as shown) (H) Error bars in all figures represent SEM.

To further determine the relevance of hBD2’s antimicrobial effects on prevention of experimental NEC, we used our recently described modification of the Paneth cell disruption NEC model with formula gavage replacing *K. pneumoniae* exposure as the second hit of the model.^57^^,^^58^^,^^64^ Thus, this model does not use the addition of exogenous live bacteria to induce disease. In brief, P14-P16-day-old C57Bl/6J mice were fed a prepared rodent milk substitute formula (RMS)^57^^,^^58^^,^^65^ 1 h before receiving an intraperitoneal injection with dithizone as above, and then every 3 h for a total of four feeds. hBD2 was given as a subcutaneous injection 1 h following the second gavage of RMS formula. Twelve hours following the first RMS feeding, mice were euthanized, and their intestines were examined for NEC-like injury as above. Animals exposed to RMS and dithizone had significantly increased injury scores compared to single insults alone or sham controls (*p* = 0.003, *n* = 10–16 per group). However, SQ hBD2 failed to protect against experimental NEC in this model (Figure 5A). To understand why hBD2 failed to protect against RMS NEC, we next examined the microbiome composition, and ileal gene regulation of several key cytokines. Despite both models resulting in histologic pathology consistent with NEC (sample histology is shown in Figure 5B), the relative composition of cecal bacterial phyla is significantly different between the dithizone-*Klebsiella*-induced Paneth cell dysbiosis NEC model (PC dysbiosis) and the dithizone-RMS NEC model (RMS NEC) (Figure 5C). While the NEC model involving induction of bacterial dysbiosis through gavage of *K. pneumoniae* induces significantly greater relative abundance of Proteobacteria, the RMS NEC model induces significantly greater relative abundance of Firmicutes and Bacteroidetes species (*n* = 7 Paneth NEC, 5 RMS NEC, *p* = 0.01 Bacteroidetes, 0.007 Firmicutes, and <0.0001 Proteobacteria). Notably, subcutaneous hBD2 delivery in our RMS NEC model did not significantly impact the microbiome of NEC animals at the phylum or family levels, which is similar to observations from hBD2 treatment in the Paneth cell disruption with bacterial dysbiosis NEC model (Figures 5D–5F). Further, the gene regulation signatures of the two models slightly differ. While the Paneth cell disruption with bacterial dysbiosis NEC model induced an upregulation of IL-1b and TNF genes, the RMS NEC model induced IL-6 and KC/GRO genes (*n* = 3–4 per group, *p* values as shown) (Figure 5G). While hBD2 did not impact injury in the RMS NEC model, it did have a significant impact on serum cytokine levels. The induction of experimental NEC through the RMS NEC model caused significant increases in serum levels of IL-1b, IL-6, KC/GRO, and TNF compared to both sham treatment and RMS exposure alone (n = 5–10 per group, *p* values as shown). However, subcutaneous treatment with hBD2 significantly decreased all four cytokines (Figure 5H).

### hBD2 improves epithelial restitution and tight junction expression

**Figure 6:**
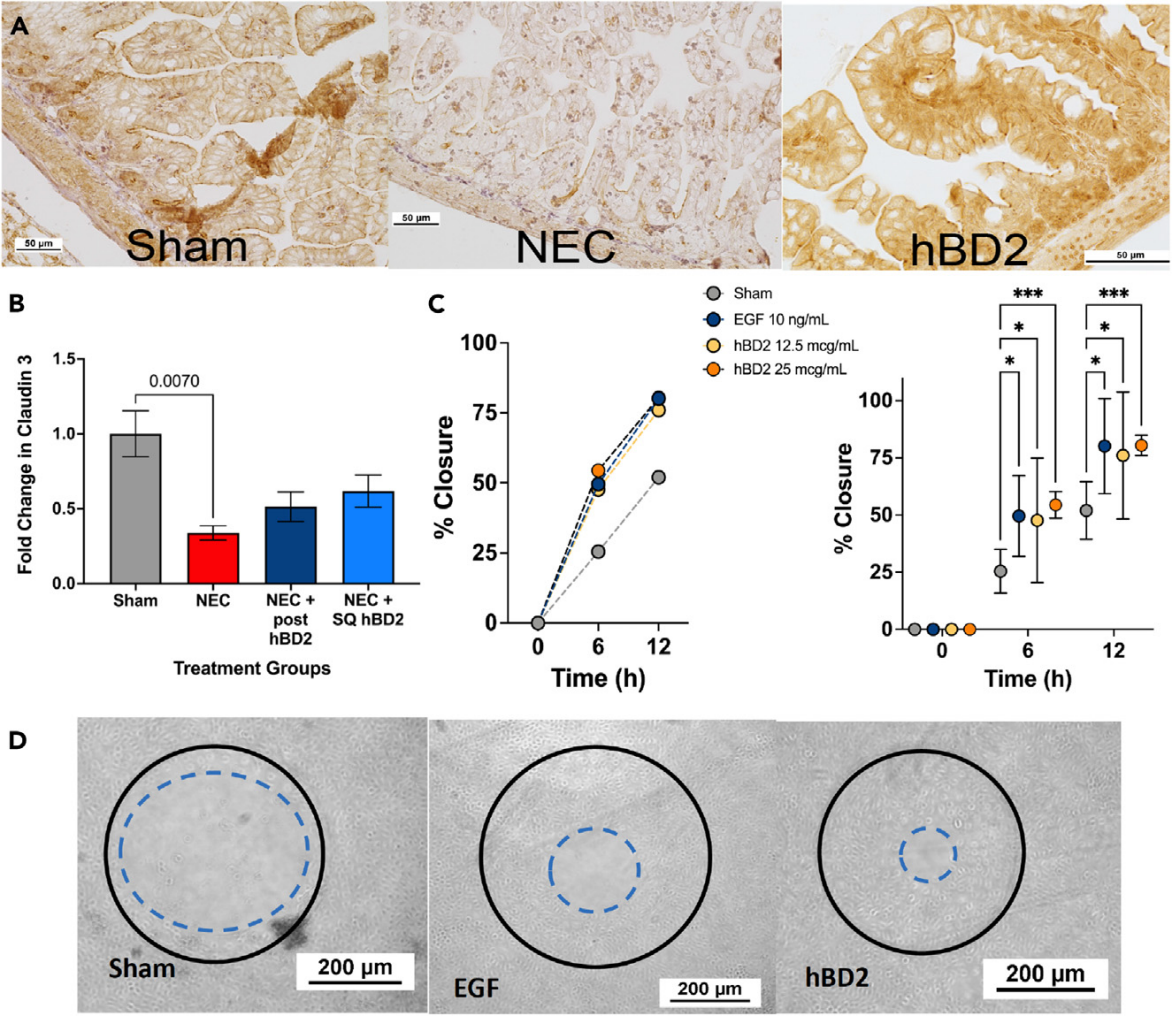
hBD2 improves epithelial restitution and tight junction expression Intestinal samples were harvested from P14-P16 mice following NEC induction with or without treatment with subcutaneous hBD2 and compared to sham animals for ZO-1 expression by immunohistochemistry. NEC induced visual loss of intestinal architecture and ZO-1 expression compared to sham and was partially recovered by hBD2 treatment. Representative samples shown at 20× with ZO-1 stain (brown) shown, *n* = 5 animals per treatment (A). Intestinal samples were homogenized and quantified for Claudin 3 by Western blot analysis. NEC significantly decreased Claudin 3 expression compared to sham controls (*p* = 0.007, *n* = 4), while hBD2 treated NEC animals were not statistically different from sham controls (B). IEC-18 monolayers were wounded with a rotating silicone disk and treated with 12.5 μg/mL or 25 μg/mL of hBD2 and compared to treatment with 10 ng/mg EGF and sham control. Both hBD2 doses and EGF showed significantly increased wound closure at 6 (48%, 54%, and 50% respectively) and 12 h (76%, 80%, and 80%) compared to sham controls (25% and 52%) (*p* = 0.02, *n* = 9 wounds per condition) Left panel shows closure curves, Right panel shows individual points with statistics and SEM. For all *p* values, ∗ ≤0.05, ∗∗ ∗ ≤0.01, ∗∗∗ ≤0.001, and ∗∗∗∗ ≤0.0001 (C). Representative micrographs taken at 40 × 6 h after wounding. The initial wound size can be seen as the solid line, while the leading edge of the wound was marked using NIS elements software and is noted by the dotted line (D).

While NEC is a multifaceted disease process, epithelial processes such as tight junction biology and cellular migration and proliferation are central for this disease. Thus, we next examined the impact of hBD2 on the immature intestinal epithelia. To determine the impact of hBD2 on tight junction expression, intestinal samples were obtained from mice treated with SQ hBD2 following intestinal dysbiosis NEC induction and compared to NEC only animals and sham controls for ZO-1 expression (Figure 6A). Induction of NEC led to a loss of ZO-1 expression compared to shams and hBD2 treatment prevented this loss (*n* = 5 for each group). To verify this loss, we also examined homogenized ileal samples from the same groups by western blot analysis for quantity of the tight junction protein Claudin 3 (Figure 6B). NEC induction significantly decreased Claudin 3 by 65% compared to sham controls (*p* = 0.007, *n* = 4), while SQ hBD2 treatment showed partial protection from NEC-induced Claudin 3 loss (38% decrease from sham, *p* = 0.4991 *n* = 4). To further understand the impact of hBD2 on epithelial restitution, we wounded rat intestinal ileal epithelial IEC-18 cell monolayers using a rotating silicone disk.^58^^,^^66^^,^^67^ Monolayers were then treated with 12.5 μg/mL or 25 μg/mL hBD2 and compared to treatment with 10 ng/mL epidermal growth factor (EGF)^83^ and sham control (Figures 6C and 6D). No cellular death was observed with either hBD2 treatment. Both hBD2 doses and EGF showed significantly increased wound closure at 6 (48%, 54%, and 50% respectively) and 12 h (76%, 80%, and 80% respectively) compared to sham controls (25% and 52%) (*p* ≤ 0.02, *n* = 9 wounds per condition). LL-37 has also been shown to aid in wound closure at low concentrations.^31^^,^^68^^,^^69^ To better compare the two HDPs, LL-37 was also examined using the wound healing assay. While the low concentration of LL-37 (1 μg) resulted in improved wound healing compared to sham control (*p* < 0.001) and even higher than that of EGF by 24 h post treatment, higher concentrations of LL-37 (12.5 and 25 μg) resulted in significantly decreased wound healing compared to sham (*p* ≤ 0.009) at both the 12- and 24-h time points (Figure S3). Collectively, these data suggest that hBD2 positively modulates components of barrier function without significant cellular damage in the immature intestinal epithelia compared to LL-37, which displayed slower wound healing with the low dose, but decreased wound healing at higher concentrations.

## Discussion

NEC continues to be one of the most devastating intestinal disorders seen in premature infants.^23^ Currently available treatment strategies have displayed limited ability to improve the high rates of morbidity and mortality associated with the disease, which has driven research into other potential therapeutics.^15^^,^^24^ Endogenous HDPs, including cathelicidins and defensins, play an important role in intestinal defense,^27^^,^^28^^,^^29^^,^^30^^,^^31^ making the exogenous use of HDPs an attractive therapeutic possibility. However, while limited, previous HDP studies in neonatal murine models have shown that not all HDPs are created equal. Studies in murine NEC models with the exogenous defensin human beta defensin-3 (hBD3) showed some decrease in disease severity but also showed concerning cytotoxic effects in *in vitro* culture. In addition, prior studies in murine sepsis models have shown that LL-37 can be dose-dependent between therapeutic and toxic levels. Lastly, while the antimicrobial effects of HDPs are a defining trait, the potency varies between HDPs and there are also many known instances of HDPs having beneficial non-antimicrobial activities.^43^ Thus, there is a significant gap in knowledge regarding the potential therapeutic impact of HDPs on NEC. Both LL-37 and hBD2 are expressed by intestinal epithelial cells and have been shown to have antimicrobial, anti-inflammatory, immunomodulatory, and wound healing effects in other studies.^27^^,^^28^^,^^29^^,^^30^^,^^31^ In this study, we hypothesized that both HDPs would provide protection from experimental NEC through various mechanisms of action, including antimicrobial properties, immunomodulatory properties, and wound healing effects. Importantly, these three endpoints are all dysregulated in our lab’s NEC models.^57^^,^^58^^,^^60^^,^^61^^,^^64^^,^^70^ Our data show that hBD2 is safe as an *in vivo* treatment in newborn mice with immature intestines. We also demonstrate that oral hBD2 given either before or after NEC induction as well as subcutaneous injection of hBD2 and oral LL-37 after NEC induction significantly decreased NEC injury scores. Supporting our hypothesis, different mechanisms of action were identified. LL-37 seems to function primarily through antimicrobial mechanisms and secondarily functions through improving wound healing at low concentrations. In contrast, hBD2 seems to have more limited antimicrobial activity and instead impacts the microbial/host axis, the host immune system, and the host epithelial layer. Together, these new data show the promise of using HDPs as a therapeutic approach in the management of NEC.

Unlike other human beta defensins, including hBD3, hBD2 appears to have limited cytotoxic effects (Figure 1).^71^ Similar to hBD2, hBD3 possesses antimicrobial activities, regulates innate immune mechanisms, attenuates or enhances chemokine and pro-inflammatory cytokine responses, impacts epithelial layer homeostasis, and enhances adaptive immune responses. However, hBD3 has significant cytotoxicity at higher concentrations in human erythrocytes,^72^ monocytes,^73^ dendritic cells, and keratinocytes.^74^ Data regarding the use of human beta defensins or cathelicidins as treatment for NEC-like injury in animal models are sparse. Sheng et al. used hBD3 in the hypoxia/formula rat model of NEC-like injury and were able to significantly decrease tissue inflammation and NEC-like damage, presumably by interrupting signal pathways downstream of TLR4.^75^ However, while they found no *in vivo* toxicity using recombinant hBD3 in rat pups, the dose curves were not provided despite disturbing data indicating dose-dependent cellular death *in vitro*. Thus, study of other defensins with low *in vivo* toxicity such as hBD2, offers the potential for therapeutics especially for susceptible premature infants.^56^ Koeninger et al. demonstrated in adult mice that the only deleterious side effect of subcutaneous hBD2 was pruritus, and this was only with doses which far exceeded the identified therapeutic doses.^56^ While our data similarly showed only minor intestinal injury with increasing doses of oral hBD2 (Figure 1) and no cytotoxicity to intestinal epithelial cells in the wound healing assay (Figures 6C and 6D), LL-37 had high cytotoxicity in our wound healing assay at concentrations of 12.5 μg/mL or higher (Figure S3). Together our results suggest that hBD2 may be a superior therapeutic HDP for impacting intestinal tract biology, especially in more susceptible populations such as preterm infants.

As mentioned above, bacterial dysbiosis is one of the hallmarks of NEC and our bacterial dysbiosis NEC model mimics this through the gavage of *K. pneumoniae*.^57^^,^^60^^,^^70^^,^^76^^,^^77^^,^^78^ Both LL-37 and hBD2 have been shown in multiple publications to have various mechanisms of action, but their broad-spectrum antimicrobial properties have been widely acknowledged.^31^^,^^44^ Our data demonstrates that LL-37 could both kill and limit the growth of our lab’s strain of *K. pneumoniae* (Figure 4E); however, our data suggest a more complex effect of hBD2 in prevention of NEC than just through its antimicrobial activities. Our data demonstrate a decline in the amount of ileal *K. pneumoniae* DNA when hBD2 was given as a treatment through oral or subcutaneous delivery routes in a similar manner to treatment with oral LL-37 (Figure 4A), which would support an antimicrobial effect. Additionally, we observed no impact of hBD2 on injury scores in our formula NEC model (Figure 5A), which does not utilize gavage of *K. pneumoniae*. However, we were unable to achieve an MIC against *K. pneumoniae* using hBD2 in either of the two different *in vitro* MIC methodologies utilized (Figures 4B and 4C). Furthermore, we did not observe a significant impact on the cecal relative abundance of Proteobacteria or more specifically Enterobacteriaceae when hBD2 was given, and we saw no significant impact on the alpha or beta diversity between NEC animals and NEC animals given hBD2 (Figure 3). There are several possible explanations for these discrepancies. First, while studies have shown effective killing of *K. pneumoniae* by hBD2, our data showed ineffective activity particularly in the NB media used for culture. This may be due to higher salt concentrations in the NB compared to TSB as it is well documented that many HDPs, including hBD2 are sensitive to salt as it interferes with hBD2’s cationic capacity.^79^^,^^80^ Second, it is likely that our NEC and *in vitro* models are utilizing complimentary, but divergent mechanisms to induce injury. Third, while next generation sequencing of the microbiome is able to identify DNA from organisms that are present, it is unable to determine whether those organisms are alive or dead. Lastly, it is possible that hBD2 may be working synergistically with another HDP or molecule in the intestine as has been previously described.^29^^,^^31^^,^^81^

While the exact pathophysiology of NEC remains elusive, the immature gut has altered apoptosis and immature tight junction proteins leading to increased intestinal permeability which likely plays a role in NEC pathogenesis. The impact of β-defensins on intestinal permeability has not been well characterized, although porcine β-defensin 2 has been shown to upregulate tight junction proteins and decrease permeability to FITC dextran in a DSS rodent model.^82^^,^^83^^,^^84^ Our data are consistent with these findings as hBD2 treatment significantly improved percent wound healing *in vitro* and tight junction expression compared to animals induced with NEC alone *in vivo* (Figure 6). In contrast to β-defensins, LL-37 has been well documented to activate cell migration, decrease epithelial permeability, and stimulate angiogenesis in different mucosal surfaces such as the airway or gastrointestinal tract.^71^^,^^85^^,^^86^^,^^87^^,^^88^ As mentioned, many of the epithelial mechanisms that LL-37 can impact are dysregulated in NEC. Importantly, while our data support the wound healing effect of LL-37 specifically at low doses, the high dose toxicity of LL-37 is concerning with respect to use in preterm infants (Figure S3). Additionally, it is notable that hBD2 was able to improve wound healing at both doses tested faster than that of LL-37. Because NEC onset can occur rapidly, having a treatment strategy that can work quickly is imperative.

In conclusion, hBD2 can significantly reduce intestinal injury in an experimental mouse model of NEC when given either before or after NEC induction and LL-37 can reduce intestinal injury when given after NEC induction. These data are the first *in vivo* proof of these HDPs’ therapeutic efficacy in treating and preventing experimental NEC. Further, our data show that hBD2 is acting on the immature intestinal milieu through several mechanisms while supporting no off-target side effects. While LL-37 also is acting through several mechanisms, high concentrations appear to have cytotoxic effects, which make it less appealing for use in susceptible premature infants. We speculate that hBD2 may represent a therapeutic or prophylactic treatment for premature infants who are vulnerable to NEC. Further studies are needed to fully characterize the mechanism of action of hBD2 particularly regarding the immunological mechanisms hBD2 may be working through in preventing and treating NEC.

### Limitations of the study

In our study, we did not specifically examine sex-specific effects. In prior publications with our NEC models, we have not found any sex-specific differences, nor have there been any reported in other literature for NEC models, hBD2, nor LL-37. We also limited our studies to only C57Bl6/J mouse strains for the purpose of tighter experimental control. We acknowledge that different strains may respond differently to HDPs. An added limitation to this study is that we are unaware of published literature demonstrating the amount of HDPs produced in our murine neonatal NEC models. Mice do not produce hBD2 or LL-37 inherently. Instead, they produce murine beta defensin 3 (mBD-3) and cathelicidin related antimicrobial peptide (CRAMP), respectively. Thus, it is unknown what the inherent HDP levels are in the murine gastrointestinal tract during an NEC exposure and how the concentrations of exogenous HDP we provided in these experiments compare. Further, there has been literature demonstrating that hBD2 retains antimicrobial activity after gavage and subsequent exposure to the gastric acid pH in the context of graft versus host disease and LL-37 is expressed in the stomach in response to *Helicobacter pylori* and retained antimicrobial activity after exposure to acidic pH.^89^^,^^90^ However, it is unknown in the context of the NEC models utilized in this study how much antimicrobial activity the HDPs retain after being exposed to stomach acid. Based on the 16S microbiome analysis, limited microbiome impact was observed after exposure to hBD2 and LL-37, but 16S sequencing is unable to distinguish what bacteria are alive versus dead. Overall, our findings highlight some of the mechanisms of action of HDPs aside from being antimicrobials. Future studies should be performed to address some of the questions remaining about the antimicrobial capacity of the HDPs utilized in this study and how they compare or interact with murine HDPs.

## STAR★Methods

### Key resources table

**Table:** 

REAGENT or RESOURCE	SOURCE	IDENTIFIER
**Antibodies**		

Anti-Claudin 3 Primary Antibody	Abcam	52231
Anti-Rabbit	Cell Signaling Technologies	7074

**Bacterial and virus strains**		

*Klebsiella pneumoniae* 10031	ATCC	PCl 602
*Escherichia coli* 47076	ATCC	MG1655
*Bifidobacterium adolescentis*	German Collection of Microorganisms and Cell Cultures GmbH	DSM20083
*Streptococcus thermophilus*	German Collection of Microorganisms and Cell Cultures GmbH	DSM20317
*Bifidobacterium breve*	Ardeypharm	

**Biological samples**		

Epidermal Growth Factor (EGF)	PeproTech	315–09
RMS Formula	Dvorak et al.^65^	
Pedialyte	Abbott	00336

**Chemicals, peptides, and recombinant proteins**		

LL-37	Sigma Aldrich	94261
hBD2	Defensin Therapeutics	

**Critical commercial assays**		

Meso-Scale Discovery V-Plex assay	Meso-Scale	Mouse cytokines
Quant-iT dsDNA High sensitivity Assay Kit	Invitrogen	Q33120
hBD2 ELISA kit	Phoenix Pharmaceuticals	EK-072-37
LL-37 ELISA kit	Hycult Biotech	HK321

**Experimental models: Cell lines**		

Intestinal Epithelial Cells (IEC)-18	ATCC	CRL-1589

**Experimental models: Organisms/strains**		

Mouse: C57Bl/6J	Jackson Laboratory	JAX: 000664

**Oligonucleotides**		

Pf (5′-ATT TGA AGA GGT TGC AAA CGA T-3′) and Pr2 (5′-CCG AAG ATG TTT CAC TTC TGA TT-3′)	Integrated DNA Technologies Liu et al.^63^	

**Software and algorithms**		

QIIME 1.9.1	QIIME Caporaso et al.^95^	
Greengenes 16S rRNA database	Second Genome DeSantis et al.^99^	
PyNAST	QIIME Bokulich et al.^96^ Caporaso et al.^100^	
EMPeror PCoAs	EMPeror Vázquez-Baeza^102^	
Chao1	Hughes et al.^103^^,^ Chao et al.^104^	
ImageJ	National Institute of Health	
Nikon Image Software (NS Elements)	Nikon	

**Other**		

ZR Fecal DNA MiniPrep Kit	Zymo Research	D6010
ZymoBIOMICS 96 Magbead DNA Kit	Zymo Research	D4308
QIAquick PCR Purification Kit	Qiagen	28104

### Resource availability

#### Lead contact

Further information and requests for resources and reagents should be directed to and will be fulfilled by the lead contact, Steven McElroy (sjmcelroy@ucdavis.edu).

#### Materials availability

This study did not generate new or unique reagents.

#### Data and code availability

Data generated to support the findings of this study are available from the lead contact on request. No original code or other similar items were utilized to generate the findings of this manuscript. Any additional information required to reanalyze the data reported in this paper is available from the lead contact upon request.

### Experimental models

#### Mice

All animal experiments were performed according to protocols approved by the University of Iowa Institutional Animal Care and Usage Committees (#8041401). C57Bl/6J mice were used whose founders were purchased from Jackson Laboratories. All mice were housed and bred under standard conditions in an AAALAC-approved vivarium. All mice were dam-fed prior to experiments, and unless otherwise indicated, experiments were conducted with male and female P14–16 mice, apart from safety experiments which were also performed on P7 mice. On the day of experimentation, animals were separated from their mothers, littermates were randomly assigned to an experimental group, and then were maintained in a temperature- and humidity-controlled chamber.

#### Induction of standard Paneth cell dysbiosis NEC model

NEC like injury was induced using the Paneth cell disruption/bacterial dysbiosis model.^60^^,^^61^ P14-16 mice were given an intraperitoneal injection with either 75 μg/kg dithizone (Sigma) dissolved in 25 mM Lithium bicarbonate solution, or an equivalent volume of Lithium bicarbonate solution alone.^61^ Six hours after injection, mice were given a gastric gavage of 1 × 10^8^ CFU/g body weight of *K. pneumoniae* 10031 (ATCC, Manassa, VA) or an equivalent volume of sterile media (nutrient broth; ATCC).^60^ Mice were monitored for 10 h after gavage and then euthanized for tissue harvesting. Mice were kept separate from their dams during the experiment.

#### Induction of RMS formula NEC model

NEC-like injury was induced using a model described in Lueschow et al. 2020.^57^ Briefly, P14-P16 mice were given an intraperitoneal injection with either 75 μg/kg dithizone (Sigma) dissolved in 25 mM Lithium carbonate solution, or an equivalent volume of Lithium carbonate solution alone. One hour before injection with dithizone and then every 3 h after the initial feed for a total of four feeds, mice were given a gastric gavage of 200 μL rodent milk substitute (RMS) formula or an equivalent volume of Pedialyte (Abbott) as a control.^57^^,^^58^^,^^65^ Mice were monitored for the duration of the 12-h experiment and then euthanized for tissue harvesting. Mice were kept separate from their dams in temperature and humidity-controlled incubators for the entirety of the experiment.

#### Bacteria

All intestinal dysbiosis NEC model studies were performed using *Klebsiella pneumoniae* (*K. pneumoniae*) 10031 (ATCC, Manassas, VA). Prior to gavage or experiment, all bacteria were grown to log-phase and optical density (OD) 600 was performed to determine colony forming unit (CFU) quantity. All mice receiving *K. pneumoniae* were given 1 × 10^8^ CFU/g body weight via single gavage feed. *E. coli* MG1655 for the MIC’s was obtained from Dr. Craig Ellermeier’s lab, University of Iowa. Bacterial strains for the turbidity assay including *Bifidobacterium adolescentis* DSM20083 and *Streptococcus thermophilus* DSM20617 were obtained from German Collection of Microorganisms and Cell Cultures GmbH (Braunschweig). *Bifidobacterium breve* was provided by Ardeypharm (Germany).

#### LL-37

LL-37 (Sigma Aldrich) 100 μg/kg body weight was administered by gastric gavage twice daily for three days prior to induction of NEC to determine whether LL-37 could prevent intestinal injury and inflammation associated with experimental NEC in prevention studies. In separate treatment studies, LL-37 was administered 1 h following *K. pneumoniae* administration to determine if LL-37 could be used as treatment to decrease the intestinal injury and inflammation seen in our animal model of NEC.

#### Human beta Defensin-2

hBD2 was provided by Defensin Therapeutics (DT). Production and purification of recombinant hBD2 was done via previously described methods.^56^ To determine the safety profile of hBD2, P7 and P14 – P16 C57Bl/6J mice were given gastric gavage of 0.1 mg/kg, 0.3 mg/kg, 0.6 mg/kg, 0.9 mg/kg, or 1.2 mg/kg twice daily for two days. Serum samples, intestinal tissue samples, and cecal stool samples were obtained as described below. To investigate the potential of hBD2 to prevent or treat NEC, mice were divided into three test groups: hBD2 prevention (pre), hBD2 treatment (post), and hBD2 subcutaneous (SQ). In the prevention group, C57Bl/6J mice were given gastric gavage of 0.6 mg/kg of hBD2 twice daily for three days prior to induction of NEC. In the treatment group, gastric gavage of 1.2 mg/kg of hBD2 was given to C57Bl/6J mice 1 h after *K. pneumoniae* gavage. In the subcutaneous hBD2 group, C57Bl/6J mice were given subcutaneous injection of hBD2 (0.3 mg/kg) into the nape of the neck 1 h after *K. pneumoniae* gavage.

#### Cells

For wound healing assays, intestinal epithelial cells (IEC)-18 (ATCC) were cultured in Dulbecco’s modified Eagle medium (DMEM) with 10% fetal bovine serum at 37°C with 5% CO2 for 24 to 48 h until a lawn developed. As these cells are commercially available, no further authentication was performed. We did not routinely test cell cultures for mycoplasma.

### Method details

#### Serum collection

Prior to euthanasia, blood was obtained from the submandibular (facial) vein as previously described and collected into a 1.5 mL Eppendorf tube (Eppendorf, Hamburg, Germany).^91^ Whole blood samples were placed on ice for 1 h then centrifuged at 5478 rcf for 5 min to isolate serum. Cytokines were quantified using a Meso-Scale Discovery V-Plex assay (Meso-scale, Gaithersburg, MD) according to the manufacturer’s instructions. Plates were read on a Sector Imager 2400 at 620 nm.

#### Microbiota composition analysis

Cecal microbial analysis was performed as previously described.^70^^,^^92^ In brief, ceca were removed and stored at −80°C until processing. For one set of samples, the ZR Fecal DNA MiniPrep kit (Zymo Research, Irvine, CA) was used to extract DNA from the intact ceca, and extracted DNA was stored at −20°C. For a second set of samples DNA was extracted using the ZymoBIOMICS 96 Magbead DNA Kit (Zymo Research, Irvine, CA), adapted from the ThermoFisher Scientific Kingfisher Flex Platform (Waltham, MA). Extracted DNA was quantified using the QuantIT dsDNA kit, High Sensitivity (Waltham, MA). 16S rDNA amplification and sequencing were performed as previously described^92^^,^^93^^,^^94^ using the Earth Microbiome Project standard protocols (www.earthmicrobiome.org) using the V4 domain and the following primers: F515 (5′-*NNNNNNNN*GTGTGCCAFCMGCCGCCGCGGTAA-3′) and R806 (5′-GGACTACHVGGGTWTCTAAT-3′), with the forward primer modified to contain a unique 8 nucleotide linker sequence (italicized poly-N section of the primer above) and a 2-nucleotide linker sequence (bold, underlined portion) at the 5′ end. PCR reactions used 5–100 ng DNA template, 1× GoTaq Green Master Mix (Promega, Madison, WI), 1 mmol/L MgCl2, and 2 pmol of each primer. PCR was performed at 94°C for the initial 3 min followed by 35 cycles of 94°C for 45 s, 50°C for 60 s, and 72°C for 90 s, with a final extension of 72°C for 10 min. PCR amplicons were grouped at approximately equal amplification intensity ratios and were purified using the Qiaquick PCR purification kit (Qiagen, Hilden, Germany). The PCR amplicons were submitted to the UC Davis Genome Center DNA Technologies Core for Illumina paired-end library preparation, cluster generation, and 2x300 bp paired-end Illumina MiSeq sequencing. Data from the sequencing run was analyzed using the QIIME software package (University of Colorado, Boulder, CO, version 1.9.1).^95^ Sequences were quality filtered and demultiplexed, and then UCLUST (drive5.com, Tiburon, CA) was used to assign operational taxonomic units (OTUs) to the sequences, based on a 97% pairwise identity.^96^^,^^97^ Secondary filtration of 0.005% was used to remove low-abundance OTUs.^96^ The filtered OTUs were taxonomically classified based on the Ribosomal Database Project classifier (Michigan State University, East Lansing, MI)^98^ against a representative subset of the Greengenes 16s rRNA database (Second Genome, South San Francisco, CA, gg_13_5 release).^99^ OTU sequence alignment was performed using PyNAST (University of Colorado)^96^^,^^100^ and was used to construct a phylogenetic tree for β diversity analyses. Relative abundances of the taxa were calculated based on a combination of data from the OTU classification as well as the frequency counts of the various OTUs from the UCLUST step. β diversity was estimated by calculating unweighted and abundance weighted UniFrac distances.^101^ Sample clustering was based on between-sample distances. EMPeror weighted UniFrac principal coordinate analyses (PCoAs) were generated to examine the beta diversity of samples.^102^ Alpha diversity analysis was performed and chao1 was used as a measure for species richness.^103^^,^^104^

#### Klebsiella pneumoniae specific primer PCR

After completion of NEC model runs, small intestinal ileal tissue was harvested and stored in RNA later (Invitrogen, Waltham, MA) overnight at 4°C. Tissue was then blotted dry and stored at −20°C until ready for DNA extractions using a ZR Fecal/Soil DNA MiniPrep kit (Zymo Research, Irvine, CA). Extracted DNA was then measured for concentration using a Nanodrop 2000 (Thermo Scientific, Waltham, MA) and stored at −20°C until ready for PCR. Three samples per treatment group were chosen for PCR and the samples were all normalized to the lowest DNA concentration from the sample set to prevent skewing of *Klebsiella* levels. *K. pneumoniae* specific primers^63^ Pf (5′-ATT TGA AGA GGT TGC AAA CGA T-3′) and Pr2 (5′-CCG AAG ATG TTT CAC TTC TGA TT-3′) were used for amplification with the following master mix per sample: 2.5 μL 10x buffer, 0.2 μL 10 mM dNTPs, 0.5 μL of both the forward and reverse primers, 0.12 μL Taq and then brought to 24 μL per sample with water (20.18 μL). One microliter total of DNA or DNA with water to dilute to a normalized concentration was added for a total reaction size of 25 μL. The following PCR parameters were utilized: 10 min at 94°C followed by 35 cycles of 30 s at 94°C, 20 s at 57°C, and 20 s at 72°C and then a 10-min hold at 72°C before ending at 4°C until removal of samples from the thermocycler. Ten microliters of PCR product were then run on a 1.5% (w/v) agarose gel electrophoresis in 1xTAE buffer at 100 V. The gel was then imaged on a BioRad Gel Doc Imager (BioRad, Hercules, CA) and ImageJ (Bethesda, MD) was used to get a pixel number for the bands that were observed.

#### Minimum inhibitory concentration

For hBD2, *K. pneumoniae* was grown in Nutrient Broth (NB) (ATTC) to mid-log phase and then plated in a 96 well plate at a concentration of 6.3 × 10^6^ - 1.1 × 10^7^ CFU/mL or 3.2 - 5.5 × 10^5^ CFU/well. *E. coli* was also grown in NB (ATTC) to mid-log phase and then plated in a 96 well plate at a concentration of 2.25 × 10^6^ - 1.585 × 10^7^ CFU/mL or 1.125 × 10^5^ – 7.925 × 10^5^ CFU/well. Increasing concentrations of hBD2 (2.5 μg/mL - 160 μg/mL) were created in 2× NB (ATTC) in a 96 well plate. For LL-37, *K. pneumoniae* was grown to mid-log phase in NB (ATTC) and then plated in a 96 well plate at a concentration of 5.15 × 10^6^ - 1.18 × 10^7^ CFU/mL or 2.58 × 10^5^ - 5.88 × 10^5^ CFU/well. Increasing concentrations of LL-37 (1 μg/mL – 80 μg/mL) were created in NB. Following inoculation with bacteria and either AMP, plates were incubated at 37°C for 12 h and analyzed at optical density (OD) 600 on a SpectraMax M2 microplate reader (Molecular Devices, San Jose, CA).

#### Modified minimum inhibitory concentration

The second MIC methodology was modified from Campos et al., 2004 and other similar publications.^105^^,^^106^^,^^107^ In brief, *K. pneumoniae* was grown in NB (ATTC) to mid-log phase and then was pelleted and washed with phosphate buffered saline (PBS). The pellet was resuspended in 10 mM PBS and 1% tryptic soy broth (TSB) or NB (ATTC) to a concentration of 1 × 10^4^ - 1 × 10^5^ CFU/mL hBD2 concentrations tested included 6.25 μg/mL – 100 μg/mL. The tubes were incubated for 1 h at 37°C and then a consistent volume was spread onto LB agar plates in triplicate per experimental replicate. Colony counts were completed ten to 12 h after inoculation and then percentage survival was calculated compared to controls.

#### Turbidity assay

The tested bacteria were incubated overnight in 1× TSB broth, centrifuged, and washed with 1 mM sodium phosphate buffer containing 1% (w/v) TSB broth. 5 × 10^5^ CFU/mL bacteria were mixed with different hBD2 concentrations in 10 mM sodium phosphate buffer with 1% (w/v) TSB (final volume 100 μL) and incubated for 2 h at 37°C. Afterward, we added 100 μL 2× TSB broth and we measured the optical density at 600 nm (Spark 10M, Tecan, Austria). Bacterial growth was monitored for 48 h, growing at 37°C in an anaerobic jar.

#### Histology

Ileal samples were defined as the distal 1/3 of the intestine between the stomach and the cecum. Tissue was collected and fixed in neutral buffered 10% formalin, embedded in paraffin, and sectioned at 5 μm thickness. The samples were stained with Hematoxylin and Eosin (H&E) (structure), or Periodic Acid Schiff (PAS) and Alcian blue (Paneth cell quantification). Tissue was evaluated under Nikon Eclipse NiU microscope (Nikon, Melville, NY) by a single, blinded investigator. Over 300 villi/crypt units were evaluated in each animal. Villous height, villous area, and crypt depth were measured and quantified using ImageJ software as previously described.^60^^,^^61^ Generalized intestinal injury scores (Figure 1B) were determined via a 3-point intestinal injury scoring scale (0 = normal, 1 = mild, 2 = severe) based on degree of villi vacuolization, mucosal ulceration, lamina propria damage and presence of hemorrhage within villi as previously described.^59^ NEC injury scores were determined by using a well characterized 5-point scale where scores of 2 or greater are consistent with NEC-like injury.^57^^,^^60^^,^^61^^,^^70^

#### HDP concentration analysis

P14-P16 wild type C57Bl/6J mice were injected subcutaneously in the nape of the neck with hBD2 (0.3 mg/kg), gastrically gavaged with hBD2 (1.2 mg/kg), gastrically gavaged with LL-37 (100 μg/kg), or intraperitoneally injected with LL-37 (100 μg/kg mg/kg), or gastrically gavaged/injected with PBS as a vehicle control. Thirty minutes after injection/gavage, blood was harvested from the mice by facial vein puncture and the intestine from stomach to cecum was harvested. The intestinal sample was homogenized in a TissueLyser LT (Qiagen, Hilden, Germany) in lysis buffer (Tris-Triton buffer recipe, abcam.com/protocols) with added protease inhibitors and 5 mm magnetic beads. Bead beating was performed for 2 min at an oscillation frequency of 200 and samples were centrifuged at 5000 RPM for 10 min at 4°C. Serum was isolated from the blood as described above. Samples were examined for hBD2 concentration using an ELISA kit (Phoenix Pharmaceuticals, INC., Burlingame, CA). Samples were examined for LL-37 using an ELISA kit (Hycult Biotech, Uden, Netherlands). Samples were put into the 96 well plate in duplicate and upon protocol completion, the plate was read on a SpectraMax M2 microplate reader (Molecular Devices, San Jose, CA).

#### Wound healing assay

Intestinal epithelial cells (IEC)-18 (ATCC) were cultured in DMEM with 10% fetal bovine serum at 37°C with 5% CO2 for 24 to 48 h until a lawn developed. Cells were then washed and put in starve media, which consisted of DMEM with 0.5% fetal bovine serum and then placed back in the incubator for 48 h. Three circular wounds were then made in each cellular monolayer using a drill press with a modified silicone bit. Following wounding, the starve media was again added to the cells and they were left in the incubator for another 48 h. After completion of the starve period, the wounded cells (*n* ≥ 9) were treated with 10 ng/mL of EGF as a positive control, starve media alone as a negative control, or hBD2 at concentrations of 12.5 and 25 μg/mL. A separate set of wounded cells (*n* ≥ 16) were treated with 10 ng/mL of EGF as a positive control, starve media alone as a negative control, or LL-37 at concentrations of 1, 12.5 and 25 μg/mL. Wounds were observed and the area was calculated at 0, 6, 12, 24, and 48 h after treatment. Images were obtained using a Nikon Eclipse TS-2 microscope at 40X with Nikon Image Software (Nikon, Melville, NY).

#### Western blot assay

Ileal samples were homogenized using a TissueLyser LT (Qiagen), then cleared, and boiled as previously described.^60^^,^^108^^,^^109^ Proteins were separated by SDS-PAGE and transferred to nitrocellulose membranes. Membranes were incubated with anti-Claudin 3 primary antibody (Abcam, Waltham MA) overnight at 4°C, and incubated with secondary antibody (Cell Signaling) for 45 min.

### Quantification and statistical analysis

All experiments were performed in at least triplicate and specific sample sizes are denoted in the figure legends. Data was analyzed in GraphPad Prism version 9 (Boston, MA, USA). Data normality was determined using the Shapiro-Wilk test and then was analyzed accordingly. Parametric data was analyzed using a one-way ANOVA with Holm-Šídák’s multiple comparisons test and non-parametric data was analyzed using Kruskal–Wallis testing with Dunn’s correction for multiple comparisons. For wounding data and microbiome data comparing combined families and phyla, a two-way ANOVA was utilized with Tukey’s correction for multiple comparisons, while individual families were measured using Kruskal-Wallis with Dunn’s correction for multiple comparisons. Significance was set as *p* ≤ 0.05 for all experiments. Results shown in graphs represent the mean and standard error of the mean. Experimental groups were compared to a control group that combined all sham animals from all trials to increase power, account for day-to-day variations, and reduce animal numbers.
