# Supplementary material for: Host defense peptides human β defensin 2 and LL-37 ameliorate murine necrotizing enterocolitis
Source: iScience. 2024 May 15;27(6):109993. doi: 10.1016/j.isci.2024.109993 (PMC11154634; doi:10.1016/j.isci.2024.109993)
Supplement: Document S1. Figures S1–S3 [file mmc1.pdf]

iScience, Volume *27*

Supplemental information

Host defense peptides human β defensin 2 and LL-37 ameliorate murine necrotizing enterocolitis

Shiloh R. Lueschow-Guijosa, Amy H. Stanford, Jennifer N. Berger, Huiyu Gong, Timothy J. Boly, Benjamin A.H. Jensen, Peter Nordkild, Alexandra J. Leegwater, Jan Wehkamp, Mark A. Underwood, and Steven J. McElroy

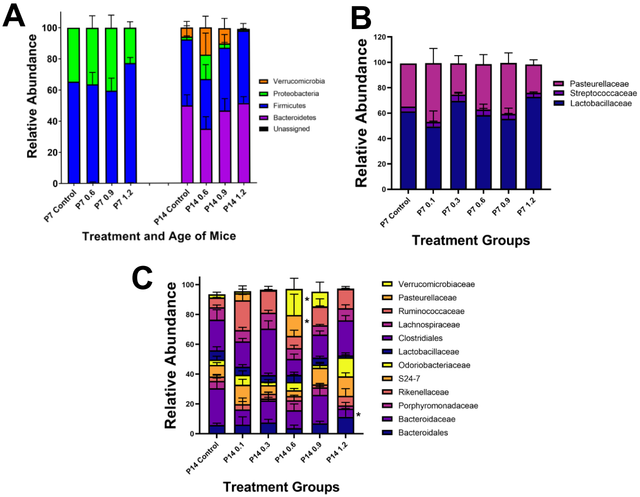

Supplemental Figure 1: hBD2 gavage does not cause biologically significant alterations in the cecal microbiome, related to Figure 1.
Increasing doses of hBD2 were given to P7 or P14-P16 C57Bl/6J mice and compared to sham for the 16S bacterial microbiome. A) hBD2 doses did not cause significant alteration in the microbiome in P7 or P14-P16 mice at the phylum level B) or in P7 mice at the family level. C) Significant family differences denoted by * were observed in P14-P16 mice given hBD2 at 0.6 mg/kg bw, but no other significant differences in P14-P16 mice given hBD2 were detected. All error bars are presented as SEM.

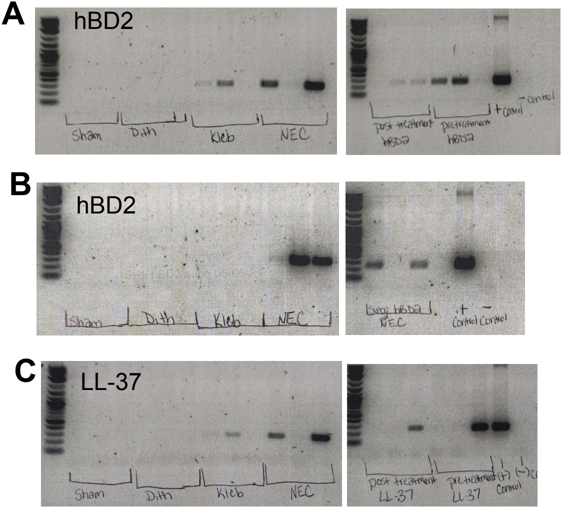

Supplemental figure 2: HDP treatment, but not pretreatment results in visual decreases in *K. pneumoniae* detected via PCR, related to Figure 4. PCR using *K. pneumoniae* specific primers on harvested ileal sections reveals varying levels of *K. pneumoniae* depending on HDP given and timing of delivery. A) Oral gavage of hBD2 model gels, B) SubQ injection of hBD2 model gels, C) LL-37 gavage model gels (n = 3 for each treatment group per blot).

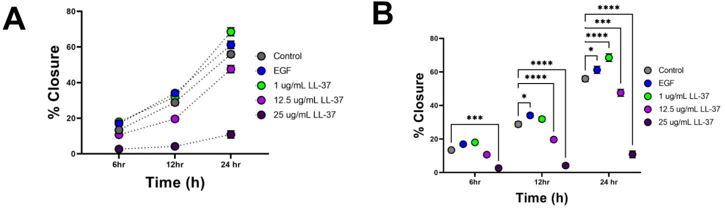

Supplemental figure 3: LL-37 improves epithelial restitution at low concentrations, related to Figure 6. IEC-18 monolayers were wounded with a rotating silicone disk and treated with 1 μg/mL, 12.5 μg/mL, or 25 μg/mL of LL-37 and compared to treatment with 10 ng/mg EGF and sham control. LL-37 at at 1 μg/mL showed significantly increased wound closure at 24 hours (69% vs control 56%, $p < 0.0001$), while EGF significantly improved wound closure compared to sham controls at both 12 (34%, $p = 0.0302$) and 24 hours (61%, $p = 0.0362$) compared to sham controls (29% and 56%, respectively) (n = 15-43 wounds per condition). 12.5 μg/mL of LL-37 resulted in significantly decreased wound closure compared to sham at both the 12- and 24 hour time points ($p \leq 0.0003$), while 25 μg/mL of LL-37 resulted in significantly decreased wound closure compared to sham controls at 6, 12, and 24 hours ($p \leq 0.0005$). A) Depicts closure curves and B) denotes individual points with statistics and SEM. For all p values, * < 0.05, ** * < 0.01, *** < 0.001, and **** < 0.0001.
